# Monitoring the burden of COVID-19 and impact of hospital transfer policies on Australian aged-care residents in residential aged-care facilities in 2020

**DOI:** 10.1186/s12877-023-04154-z

**Published:** 2023-08-22

**Authors:** Shruti Premshankar Nair, Ashley L Quigley, Aye Moa, Abrar Ahmad Chughtai, Chandini Raina Macintyre

**Affiliations:** 1 Ingham Institute, SWSLHD, Liverpool, Sydney, NSW 2170 Australia; 2 Biosecurity Research Program, The Kirby Institute, UNSW, Wallace Wurth Building, High St, Kensington Campus, Kensington, NSW 2052 Australia; 3 School of Population Health, UNSW Medicine, Samuel’s building, Kensington, Sydney, NSW 2052 Australia

**Keywords:** Residential aged-care facility, COVID-19, Hospital transfer policies, Infection control, Community transmission

## Abstract

**Background:**

Residential aged-care facilities in Australia emerged as the high-risk setting the COVID-19 outbreaks due to community transmission. The vulnerable aged-care residents of these facilities suffered due to low hospital transfers and high mortality and morbidity rates. This study aimed to monitor and report the burden of COVID-19 in residential aged-care facilities across Australia and the impact of hospital transfer policies on resident hospitalisation during the first year of the pandemic.

**Methods:**

We conducted a retrospective cohort study by collecting data from weekly aged-care outbreak reports published by open sources and official government sources between 1st March and 20th November 2020. A comprehensive line list of outbreaks was created using open-source data. The line list included the name of the facility, location, COVID-19 cases among residents, & staff, resident hospitalisations, mode of transmission, number of resident deaths, and state policies involving resident hospitalisation. We also searched the websites of these facilities to collect data on their COVID-19 policies for the residents, staff, and visitors. Statistical analyses were performed on the data obtained.

**Results:**

126 aged-care COVID-19 outbreaks were identified in Australia during the study period. The incidence rate of COVID-19 infections among aged-care residents in Australia was (1118.5 per 100,000 resident population) which is 10 times higher than the general population (107.6 per 100,000 population). The hospitalisation rate for aged-care residents in Australia was 0.93 per 100,000 population. The hospitalisation rate of aged-care residents in Victoria was 3.14 per 100,000 population despite having the highest COVID-19 cases. Excluding South Australia, all states followed ad-hoc case-by-case hospital transfer policies for aged-care residents.

**Conclusion:**

This study documented a higher risk of COVID-19 infection for aged-care residents and workers but found low hospitalisation rates among residents across Australia. The hospitalisation rates in Victoria were higher than the national average but low when considering the COVID-19 infection rates in the state. The hospitalisation rates could have been impacted due to the state hospital transfer policies at that time. Immediate transfer of infected residents to hospitals may improve their survival and reduce the risk of infection to the other residents, as healthcare settings have more advanced infection control measures and are well-equipped with trained staff and resources.

**Supplementary Information:**

The online version contains supplementary material available at 10.1186/s12877-023-04154-z.

## Introduction

As of 10th March 2023, over 676 million cases of COVID-19 have been confirmed worldwide, causing more than 6.8 million deaths [1]. Globally, high mortality rates have been found in older people residing in aged-care settings [2–4]. The risk of mortality and hospitalisation increases due to advancing age, institutionalisation, comorbidities, frailty, or too few or inadequately trained staff [5]. Moreover, the communal environment in residential aged-care facilities (RACFs) presents challenges to resident protection due to the difficulty of infection control and limited spaces for isolation or quarantine [6, 7].

The rapid spread of the COVID-19 pandemic in the RACFs is of vital concern to aged-care providers, due to rapid transmission to frail older adults due to congregate housing [5, 8]. It is well documented that visitors, residents, or staff with asymptomatic or presymptomatic infections or atypical symptoms are potential sources for SARS-CoV-2 transmission in aged-care facilities [9–11]. Many aged-care workers (ACWs) may also have barriers to healthcare access. A study found low influenza vaccination rates in ACWs, as many did not have permanent residency and access to health insurance and thus faced out-of-pocket costs for healthcare [8, 12]. ACWs are also often a low-skilled workforce with minimal training in health. Understanding potential risks associated with spread of infections in RACFs, including COVID-19, may not be well understood by aged care workers. Few ACWs are registered nurses with skilled health training in Australia [13]. Thus, the aged-care sector has been a subject of community concern in the care provided to the vulnerable population even before the pandemic [14].

In October 2019, the Royal Commission into Aged-Care Quality and Safety (Aged Care Royal Commission) found that a fundamental overhaul of design, objectives, regulation, and funding was needed. This system was expected to meet the challenges of the COVID-19 pandemic, as the Communicable Diseases Network Australia (CDNA) guidelines required RACFs to self-manage the pandemic [14, 15]. Although these guidelines were not mandatory yet, Australian RACF providers were encouraged to follow them in addition to the mandatory state and federal policies for managing COVID-19 outbreaks at their facilities. During the first and second waves of the COVID-19 pandemic, Australian RACFs struggled to mitigate the challenges presented by the rapid escalation of the COVID-19 case and high mortality rates of aged-care residents, which forced border closure, travel restrictions for domestic & international travel and complete lockdown in Australia [16].

RACF outbreaks in Victoria significantly increased between 26th May and November 2020, with 168 outbreaks [17]. The Victorian state reported a high prevalence of COVID-19 infections compared to other states and was the only State in 2020 with sustained community transmission [17]. Staff infection at a quarantine hotel in Melbourne, Victoria, initiated the second, larger epidemic that took five months to suppress. This was Australia’s largest SARS-CoV-2 epidemic in 2020, accounting for around 70% of the total cases notified in the country [17].

RACFs worldwide have adopted strategies their respective government and policymakers prescribed in infection control. During the COVID-19 pandemic, government bodies have exercised different strategies to reduce hospitalisation in acute hospitals across the globe [18]. The UK Government supported RACFs during the COVID-19 pandemic to ease pressure on acute hospitals. These facilities were instructed to accept new and returning residents despite their COVID‐19 infection status and to institute appropriate infection prevention measures. The strategy included instruction on how to ensure an adequate supply of PPE [19]. Despite these measures, RACF workers reported inadequate PPE supply in facilities that managed COVID-19-infected patients. COVID‐19 cases have occurred in over 2000 RACFs in the UK, with almost 15,000 deaths [19].

In Australia, there was controversy concerning the hospitalisation of aged-care residents during the early stage of the COVID-19 pandemic in 2020. In some cases, COVID-19-positive residents were admitted to hospitals, but in other cases, infected residents were kept on-site with “hospital-in-the-home” plans [20]. During the first wave of COVID-19 in Australia in 2020, initial guidelines issued by the CDNA advised that aged-care residents should be transferred to a hospital ‘only if their condition warrants’ it [15]. Hospital transfer of residents was advised by the Ministry of Health on a case-by-case basis [15]. However, this has since been modified, and current CDNA guidelines recommend COVID-19 risk assessment for all aged-care residents and ACWs across all states/territories. Hospital transfer is now only advised for the residents and staff categorised as high-risk [21]. The Royal Commission advised RACFs to seek medical advice through their associated general practitioners (GPs) for non-COVID hospital transfers. Excluding emergency cases, RACFs or GPs were advised to consult specialists through telehealth services wherever possible. In 2020-21, there were 697 public hospitals and 63,333 beds in Australia, and the recent data for private hospitals showed there were 657 private hospitals. Australia had 11.8 million hospitalizations (all medical conditions) during this time [22].Between January 2020 and June 2021, there were over 7300 hospitalisations involving a COVID-19 diagnosis. By 24th November 2020, there were 2007 COVID-19 hospitalisations [22].

During 2020, these highly vulnerable settings were largely left to self-manage residents amidst the COVID-19 pandemic, influenced by hospitalisation policies of State and Federal Governments. This study aimed to estimate the overall burden of COVID-19 on aged-care residents and workers in Australia during the first and second wave of the pandemic in 2020 and to assess the impact of state policies on the hospitalisation of aged-care residents across Australia.

## Methods

### Data collection

This study followed the methodology of a previous study conducted by Quigley et al. [13]. We retrospectively collected publicly available data on Australian COVID-19 outbreaks from reports generated by Federal and state/territory Governments and media sources between 1st March 2020 and 20th November 2020 to coincide with the first and second wave of the COVID-19 pandemic [23–29].

For the purposes of this study, COVID-19 cases were defined as people working or residing in aged-care with a diagnosis based on positive viral test results from respiratory swab samples. An exposure in the RACF was defined by the confirmation of a positive SARS-COV‐2 polymerase chain reaction nasooropharyngeal swab in one staff member or resident [30]. A RACF outbreak was defined as “two or more residents of a residential care facility who had been diagnosed with a COVID-19 via rapid antigen test (RAT) or polymerase chain reaction (PCR) test within 5 days and had been onsite at the residential aged care facility at any time during their infectious period” or “ five or more staff, visitors and/or residents of the residential care facility diagnosed with COVID-19 through RAT or PCR test within past 7 days who worked/visited during their infectious period [30].

A comprehensive line list of aged-care outbreaks was created by compiling data obtained from aged-care outbreak surveillance reports published by the Australian Government Department of Health and Aged Care (DHAC) available through a government report [23] and media releases/briefs released by the RACFs. Media releases/blogs of RACFs were also searched to collect the data related to hospitalisation status of aged-care residents, number of deaths, mode of transmission and the quarantine status of aged-care residents during the study period [24–29, 31, 32]. The total COVID-19 cases notification data across Australian states/territories was obtained from DHAC [33]. The frequency of hospitalisation and hospitalisation rate (HR) was calculated to assess the impact of the state hospital transfer policies on the hospitalisation of aged-care residents [20]. We assessed if the states/territories were adopting an immediate hospitalisation policy or implementing case-by-case hospital transfer policy and compared the impact of these policies on resident hospitalisation using hospitalisation rates and the frequency of COVID-19 infections as reference. Hospitalisation data for the general population was obtained from the hospitalisation data published by Australian Institute of Health and Welfare (AIHW) [22]. ACW’s were defined as any employee who worked in an aged-care setting during the COVID-19 pandemic, regardless of clinical registration. Date of COVID-19 case confirmation was recorded as a proxy for onset date.

### Data analysis

The burden of COVID-19 across Australian RACFs during the first and second wave was quantified. Incidence rates (IR) for aged-care residents and ACW were calculated by using total infections in aged-care residents and ACWs in Australia as the numerator and using total Australian aged care resident/ACW population as the denominator from aged-care resident data published by the Australian Institute of Health and Welfare https://www.gen-agedcaredata.gov.au/Resources/Access-data/2020/October/Aged-care-data-snapshot-2020 and aged-care workforce census report (https://gen-agedcaredata.gov.au/Resources/Reports-and-publications/2021/October/2020-Aged-Care-Workforce-Census-Report) released by the DoH [34, 35]. For the general population, IR were calculated by using the total COVID-19 infections in the general population as the numerator and the mid-year population as of June 2020 published by the Australian Bureau of Statistics (ABS) (https://www.abs.gov.au/statistics/people/population/national-state-and-territory-population/jun-2020) as the denominator [36].We calculated the risk ratio (RR) by comparing the overall ACW and resident cases per state with the cases reported within the general population. We also compared the overall resident deaths per state due to COVID-19 infection with the total deaths reported within the general population published by the ABS (https://www.abs.gov.au/statistics/people/population/deaths-australia/2020) as the denominator [37]. An epidemic curve was plotted to describe the distribution of aged-care cases across the nation.

We used media reports to investigate the source of infection amongst aged-care residents and workers [31, 32, 38]. The source of infection was categorized by assessing if the initial source of infection was identified, and if so if they had travelled overseas, contracted COVID-19 in a RACF, or was a local case without any confirmed epidemiological linkage. Media reports/releases by RACFs were screened to determine if the infected resident/staff acquired the infection through overseas/interstate travel, locally acquired the infection through cases within the facility with no travel history or through community transmission linked to unknown case or linked to a close contact whose source of infection is unknown. This data was presented in a graph to show distribution of sources. For each RACF identified, the number of staff in self-quarantine, number of secondary related COVID-19 infections and the facility’s policy of visitor restriction was recorded where possible, to determine the impact on the aged-care workforce. We also assessed the impact of Federal/State hospital transfer policies on resident hospitalisation.

This study was analysed and reported based on the STROBE guidelines for observational studies [39].Descriptive analysis was used to present the study data with Stata IC version 16 [40]. A Chi-square test was used to calculate the RR and for testing the significance of the deaths associated with COVID-19 cases.

## Results

There were 126 aged-care outbreaks between 1st March and 20th November 2020.

### Epidemic curve

**Fig. 1:**
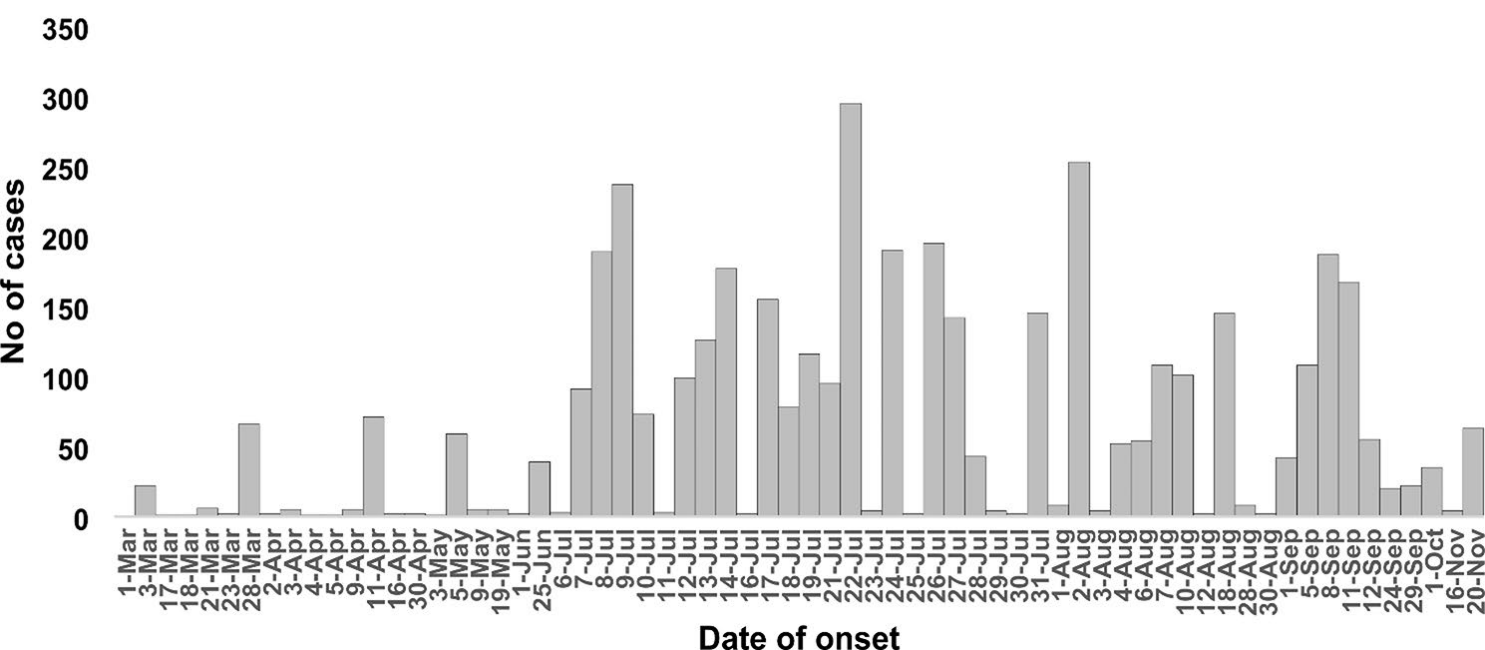
Epidemic curve of COVID-19 outbreaks in RACFs in Australia between1st March and 20th November 2020 using surveillance reports and open-source data

By 20th November 2020, 27,789 confirmed cases in the general population and 907 deaths were reported nationwide by the Australian DoH. Our study estimated 2058 COVID-19 cases among aged-care residents and 2146 ACW cases, and 585 other cases in RACFs between 1st March and 20th November 2020. Figure 1 shows an epidemic curve that describes the distribution of daily aged-care cases of COVID-19 infections and cumulative cases in Australia between 1st March and 20th November 2020. The number of daily COVID-19 cases was at its highest between 7th July and 2nd August, following which the case numbers decreased.

### Source of infection

**Fig. 2:**
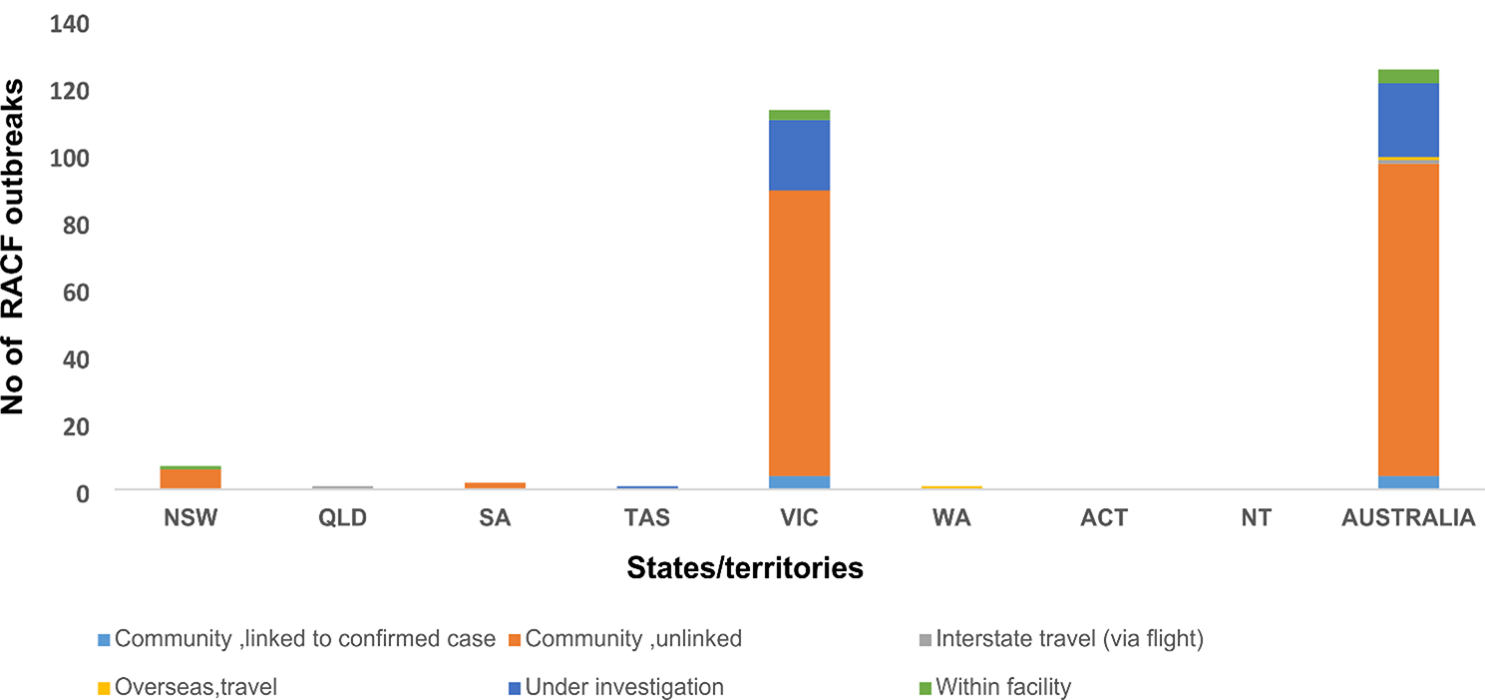
Frequency distribution of COVID-19 clusters according to their source of infections and location per States or Territory, between 1st March and 20th November 2020

Figure 2 represents the distribution of COVID-19 RACF outbreaks according to the location and source of infection. Overall, 126 RACF outbreaks were identified from open-source data, 93 outbreaks (74%) were linked to community transmission and associated with an unknown index case, 22 (17.5%) outbreaks were under investigation, 4 (3.17%) outbreaks were locally spread within the facility by staff or residents with no links to local or overseas travel, 4 (3.17%) outbreaks were traced to a confirmed case, 1 (0.79%) outbreak in Western Australia (WA) was associated with overseas travel, 1 (0.79%) outbreak was linked to interstate travel by staff and 22 (17.5%) outbreaks were under investigation at the time of this study. High levels of community transmission were reported in Victoria (VIC) during the study period.

### Analysis of COVID-19 infections of aged-care residents

As of 20th November 2020, the nationwide IR of COVID-19 infections among aged-care residents was 1118.5/100,000 resident population which is ten times higher than the general population (107.6/100,000 population). VIC reported the highest IR of COVID-19 infections among aged-care residents (3987/100,000 population), followed by NSW (93.8/100,000 population) and TAS (21.9/100,000 population). The general population had significantly lower IR in VIC (303.9/100,000 population), NSW (55.3/100,000 population) and TAS (42.6/100,000 population) compared to aged-care residents. During this period, no COVID-19 cases were reported among aged-care residents in Australian Capital Territory (ACT), South Australia (SA), WA and Northern Territory (NT).

There were 238 hospitalisations of aged-care residents according to open-source data in Australia between 1st March and 20th November 2020. The hospitalisation rate of aged-care residents affected by COVID-19 was 0.93 per 100,000 population in Australia, suggesting that only 11.6% (238/2058) of the total aged-care residents were hospitalised across the country. Victoria reported 210 hospitalisations which are 88% of the national estimate (210/238). The case-fatality rate (CFR) of RACF residents in Australia was 30.3% (624/2058) (Table 1). Our study estimates suggested 624 resident deaths in Australia (28 in NSW, 595 in VIC and 1 in TAS). Additional file 1 shows COVID-19 outbreaks in aged-care settings in Australia between 1st March and 20th November 2020.

**Table 1:** Rates of COVID-19 infections among aged-care residents per state between 1st March and 20th November 2020

State/Territories	Total COVID-19 cases^	Resident cases	IR ^#^(gen population)	IR^+^ (residents)	No of resident hospitalization	HR^#^	CFR
NSW	4517	59	55.33	97.31	27	0.33	47.5
VIC	20,345	1998	303.89	4092.25	210	3.14	29.8
ACT	115	0	26.35	0	0	0	0
TAS	230	1	42.55	22.45	1	0.18	100
SA	550	0	31.09	0	0	0	0
WA	796	0	29.90	0	0	0	0
QLD	1190	0	23.00	0	0	0	0
NT	46	0	18.70	0	0	0	0
**AUSTRALIA**	**27,628**	**2058**	**107.56**	**1118.55**	**238**	**0.93**	**30.3**

Abbreviation IR = Incidence rate; HR = Hospitalisation rate; CFR = Case fatality rate

^Total COVID-19 cases data across different states were obtained from DHAC [33]

^+^Aged-care residents’ population was chosen as denominator, AIHW-GEN Aged care data [34]. ; reported per 100,000 population .#Mid-year population was used as denominator for calculation, ABS statistics [36]; reported per 100,000 population

Analysis of the nationwide reported COVID-19 cases among aged-care residents suggested that aged-care residents were 10.5 times (95%CI 10.08–10.95) more likely to develop COVID-19 infection compared to the general Australian community, and this association was statistically significant (*p* ≤ 0.001). In VIC, the risk was 14 times higher ((95%CI 13.41–14.61, *p* < 0.001) in aged-care residents compared to the general population. In NSW, aged-care residents were 1.8 times (95% CI, 1.37–2.27, *p* < 0.001) more likely to develop COVID-19 compared to the general population of NSW. The Newmarch House RACF followed the hospital-in-home policy and residents were 1.06 times (95%CI, 0.77–1.47, *p* = 0.7079) at higher risk of acquiring COVID-19 than the general population of NSW. In TAS, aged-care residents were 0.53 times (95% CI, 0.08–3.76, *p* = 0.498) more likely to acquire COVID-19 compared to the general population of NSW, and this association was not statistically significant. Aged-care residents in Australia were 3.48 times (95%CI, 3.22–3.76, *p* = < 0.001) more likely to die due to COVID-19 infection than the general population, and this association was statistically significant. In VIC, aged-care residents were 4.82 times (95%CI, 4.45–5.52, *p* = < 0.001) at higher risk of death due to COVID-19 than the general population, and this association was also statistically significant. Aged-care residents in NSW were 0.97 times at greater risk of death due to COVID-19 infection than the general population, and this association was not statistically significant. In TAS, aged-care residents were 0.53 times (95%CI, 0.07–3.37, *p* = 0.517) at increased risk of COVID-19 death than the general population, and this association was not found statistically significant. (Table 2).

**Table 2:** Analysis of the risk of COVID-19 transmission from aged-care residents based on the Australian states/territories

State/Territory	COVID-19 infection			COVID-19 deaths		
	RR	95% CI	*p* value	*RR*	95% CI	*p* value
NSW	1.76	1.37–2.27	< 0.001* ^**#**^	0.97	0.67–1.40	**0.851**
VIC	14	13.41–14.61	< 0.001*	4.82	4.45–5.52	< 0.001*
TAS	0.53	0.08–3.76	0.498	0.53	0.07–3.75	0.517
**AUSTRALIA**	**10.51**	**10.08–10.95**	< 0.001*	**3.48**	**3.22–3.76**	< 0.001*

Abbreviation RR = Risk ratio; CI = Confidence Interval; *Statistically significant (Using χ^2^ test) ; # Risk of COVID-19 transmission from residents of the Newmarch home which followed a hospital-in-home policy (RR = 1.06; CI-0.77-1.47; p = 0.7079) ; ^a^ Calculated using the state-wide COVID-19 death notification /Total deaths in the state (excluding COVID-19) from ABS statistics [37] and COVID-19 cases in the state as source of exposure and mid-year state population as the denominator [36]

### Analysis of COVID-19 infections of ACWs

We calculated the frequency and percentage of ACW cases vulnerable to COVID-19 infections in RACFs between 1st March and 20th November 2020 (Table 3). There were 2146 ACW cases (7.8%) of the total cases in Australia. VIC recorded the highest number of ACW cases (2092, 10.3%), followed by NSW (46, 1.02%). No hospitalisation or fatality was reported among ACWs nationwide, and all cases were recovered; hence, the number of ACWs exposed to COVID-19 on quarantine/self-isolation was analysed. Our study found that 594 ACWs were under home quarantine/self-isolation in Australia; 496 ACWs on quarantine/self-isolation were reported in Victoria, followed by NSW (71). The Supplementary Material 1 highlights the policies of residential aged-care facilities regarding visitor restriction, lockdown and other mandatory guidelines, including self-isolation or home quarantine of ACWs to prevent the spread of COVID-19 infections in the facilities.

**Table 3:** Frequency distribution, percentage, and rate of infection of ACW cases vulnerable to COVID-19 infections in RACFs using open-source data between 1st March-20th November 2020 (N, %)

State/Territory	Total COVID Cases^	Total ACW cases (N, %)	Number of ACW on quarantine/self-isolation
NSW	4517	46 (1.02)	71
VIC	20,345	2092 (10.3)	496
ACT	115	0 (0)	0
TAS	230	1(0.43)	22
SA	550	5(0.90)	4
WA	796	1 (0.12)	0
QLD	1190	1(0.08)	1
NT	46	0	0
**AUSTRALIA**	**27,628**	**2146 (7.77)**	**594**

^Total COVID-19 cases data across different states were obtained from DHAC [33]

**Table 4:** Analysis of the risk of COVID-19 transmission in ACWs based on the Australian states/ territories

State/Territory	Risk ratio^#+^	95% CI	*p* value
NSW	1.86	1.39–2.48	< 0.001*
VIC	25.21	24.17–26.30	< 0.001*
QLD	0.17	0.02–1.18	0.040
WA	0.29	0.04–2.07	0.189
SA	1.30	0.54–3.11	0.555
**AUSTRALIA**	**16.66**	**15.94–17.40**	< 0.001*

# ACW population was chosen as denominator, Aged-care census report [35]

+Mid-year population was chosen as denominator for general population, ABS statistics

*Statistically significant (Using χ^2^ test)

Analysis of the nationwide reported COVID-19 cases among ACWs suggested that ACWs were 17 times (95%CI 15.94–17.40, *p* < 0.001) more likely to contract COVID-19 infection compared to the general population in Australia. ACWs in VIC were 25 times (95%CI, 24.17–26.3, *p* < 0.001) more likely to contract a COVID-19 infection than the general population. Statistically significant associations were also noted in NSW, where ACWs were 1.9 times more likely to contract COVID-19 and ACWs in QLD were 0.17 times more likely to develop COVID-19 than the general population (Table 4).

### Impact of state policies related to hospital transfer of COVID-19 on the hospitalisations rate of aged-care residents nationwide

Table 5 and Supplementary Material 1 summarise the state hospital transfer policies during the COVID-19 pandemic for aged-care residents in Australia and highlights the hospitalisation rates associated with COVID-19 and other general hospital admissions. Table 5 shows 238 aged-care resident hospitalisations (27 in NSW, 210 in VIC and 1 in TAS) according to our search between 1st March and 20th November 2020. There were 4,718 COVID-19 total hospitalisations reported across Australia. COVID-19 hospitalisations for the general population were 435 in NSW, 3,622 in VIC, 2 in QLD, 3 in WA, 61 in SA, 18 in TAS,554 in ACT and 23 in the NT. Despite high COVID-19 infection rates, the hospitalisation rate (HR) was significantly lower for aged-care residents than the general population across all Australian states and territories. The hospitalisation rate for COVID-infected aged-care residents was 0.33 per 100,000 in NSW, 3.14 per 100,000 in VIC and 0.18 per 100,000 in TAS. The HR of the general population infected with COVID diagnosis across Australia is 18.37 per 100,000 population. HRs for COVID infected general population were 0.49 in NSW, 54.1 in VIC, 0.46 in QLD, 0.55 in WA, 3.45 in SA, 0.68 in TAS, 10.71 in ACT, and 9.35 in NT. VIC reported high HR for COVID-19 infected compared to other states for the resident and general populations. HRs for other hospital admissions across Australia were 460.87 per 1000 population. HRs (all medical conditions) for the general population (per 1000 population) were 38.03 in NSW, 432.84 in VIC, 37.97 in QLD, 93.81 in WA, 459.11 in SA, 2,226.43 in TAS, 6,797.68 in ACT and 794.07 in NT. Although VIC had higher COVID-related hospital transfers than other states, the study findings confirm that only 10.5% of the total infected residents were hospitalised despite reporting the highest COVID notifications. The study findings confirm a significantly low hospital transfer rate of COVID-infected residents in NSW. Compared to the Australian 2018-19 hospitalisation data, hospital transfers have increased in 2020 despite low COVID hospitalisations [41]. This finding may suggest that hospital transfer policies could have impacted the transfer of COVID-infected residents as HRs were significantly lower for aged-care residents with COVID-19.

**Table 5:** Summary of hospital transfer policies for resident hospitalisation adopted by the Australian states/territories during the reporting period and frequency & rate of COVID and general hospitalisations across different states/territories

State/Territories	State policy guideline	No of resident hospitalization	HR^a+^	No of COVID-19 hospitalisations (gen pop)	HR^b#^	All hospitalisations	HR^c#^
NSW	Facility 1–03/03/2020-11/04/2020: All cases were transferred to hospital during the 1st outbreak. Other outbreaks: Admission to hospital was made by Ministry of Health on case-by-case basis. Residents in one of the aged-care facilities were treated at the facility under the **Hospital in the Home program** [20, 42]	27	0.33	435	0.49	3,352,532	38.03
VIC	Decision was made on case-by-case basis. [20, 43]	210	3.14	3,622	54.1	2,897,797	432.84
QLD	Residents would be transferred to hospital on case-by-case basis as recommended in the CDNA guidelines [44]	0	0	2	0.46	196,468	37.97
WA	Decision was made on case-by-case basis. [45]	0	0	3	0.55	249,722	93.81
SA	Immediate Hospital Transfer for all aged-care residents tested positive for COVID-19 [46]	0	0	61	3.45	812,296	459.11
TAS	Decision was made on case-by-case basis whether the resident/s should be managed in their home RACF or transferred to hospital [47]	1	0.18	18	0.68	1,203,608	2,226.43
ACT	Residents would be transferred to hospital on case-by-case basis as recommended in the CDNA guidelines [15]	0	0	554	10.71	2,930,480	6,797.68
NT	Residents would be transferred to hospital on case-by-case basis as recommended in the CDNA guidelines [15]	0	0	23	9.35	195,340	794.07
**AUSTRALIA**		**238**	**0.93**	**4718**	**18.37**	**11,838,243**	**460.87**

Abbreviation HR = Hospitalisation rate^+^Aged-care residents’ population was chosen as denominator, AIHW-GEN Aged care data [34]; reported per 100,000 population

#Mid-year population was used as denominator for calculation, ABS statistics [36]; reported per 100,000 population

^a^ Hospitalisation rate of aged-care residents with COVID-19 infection; reported per 100,000 population^b^ Hospitalisation rate of general population with COVID-19 diagnosis; reported per 100,000 population

^c^ Hospitalisation rate of all hospital admissions [22]; reported per 1000 population

## Discussion

Using open-source data, we estimated 2058 aged-care resident cases and 2145 ACW cases, accounting for 7.4% and 8% of the total COVID-19 infections in Australia. The numbers of COVID-19 cases reported by the Federal Government are consistent with our findings for aged-care residents though we have found slightly more cases among ACWs. This could be explained by using open-source data when official reporting was low, and there was no national RACF data reporting early in the pandemic [13]. We found low cases of ACWs from open sources compared to the official surveillance reports [23].

Current evidence on the association between outbreaks in RACFs and the incidence of COVID-19 in surrounding communities suggests that ACWs and visitors of these facilities introduce SARS-CoV-2 into such facilities [48]. The study conducted by Vivaldi et al. [53] has estimated increased odds (OR 2.4 95% CI 1.9-3.0) of COVID-19 infection of aged-care residents by ACWs who work in such facilities. Our study findings suggested that aged-care residents were 11 times and ACWs were 17 times at greater risk of contracting COVID-19 infection than the general Australian community. The infection prevention and control (IPC) guidelines for RACFs in many countries throughout the pandemic have forced RACFs to become relatively confined environments, restricting non-essential visitors and transfer of residents. The guidelines in Australia recommended that RACFs impose complete/partial lockdown during outbreaks, restricting non-essential visits /compassionate visits only, mandatory influenza vaccinations (at a time when COVID-19 vaccines were unavailable), hand hygiene and use of PPE [49]. The guidelines also acknowledge that ACWs regularly entering care homes can be sources of COVID-19 outbreaks [48].

A recent meta-analysis has found a high attack rate of 45% for COVID-19 infection, with a CFR of 23% and HR of 37% among aged-care residents [48]. The present study found that the hospitalisation rate among aged-care residents in Australia was 12%, and CFR was 30%, highlighting the role of urgent hospital transfers. Our study findings have suggested that aged-care residents in Australia were 3.5 times at higher risk of death due to COVID-19 infection than the general population. With such high infection rates, limiting COVID-19 transmission is vital [48]. Considering the high infection rate in RACFs across Australia, the Department of Health (DoH) prioritised aged-care residents and ACWs in the first phases of the national vaccine rollout [50]. This is particularly important in the recent outbreaks of COVID-19 variants of concern, such as the highly transmissible Omicron strains.

During the initial peaks of the COVID-19 outbreaks, nationwide media reports indicated overseas travel of visitors or staff as the initial cause of the outbreak in the facilities [17, 51]. Following the travel ban in March 2020, community transmission of COVID-19 cases from symptomatic and asymptomatic cases has been linked with increased COVID-19 infections [13]. Our study findings indicate that community transmission from unknown cases is most commonly the source of infection of COVID-19 outbreaks in the facilities. The Federal Government has since initiated the testing of asymptomatic cases to limit the spread of COVID-19 infection in the facilities [28]. The outbreak in the RACFs had waned by11th December 2020, but resumed in 2021 due to delta and omicron variants. As of 30th November 2022, 9,239 aged-care COVID-19 outbreaks had occurred since the beginning of the pandemic, with 101,829 residents and 63,801 ACW cases [23]. SARS-CoV-2 virus containing a D614G substitution in the spike glycoprotein was the predominant circulating variant during Australia’s first and second wave of the COVID-19 pandemic, but far more contagious variants now prevail [52, 53].

The Aged care Royal Commission found the most common reasons for hospital admissions and emergency department presentations before the COVID-19 pandemic (2018–2019) among aged-care residents (≥ 65 years old) were respiratory diseases, injuries, circulatory diseases, dialysis, and ‘symptoms and signs‘ [41]. The report suggested that 31.1% of residents were admitted to a public hospital at least once (increasing to roughly 37% when private hospital admissions are included). This report also mentioned that 36.9% of residents presented to an emergency department at least once [41]. The present study indicates high hospitalisation rates for the general population despite COVID notifications in the state. However, we cannot confirm the same for aged-care residents during the pandemic, as residents were transferred to hospitals on a case-by-case basis across all states, excluding SA for COVID infection. Since there is no publicly available data on the non-covid hospital transfers of aged-care residents during the pandemic, we cannot estimate if the other hospital admissions were also affected due to the non-covid hospital transfer policy.

A Victorian population-based epidemiological study reported 168 outbreaks involving 1959 resident cases and 647 resident deaths in Victoria between 26th May 2020 and November 2020. This study reported 18,703 COVID cases until 27th November 2020 and found 3,622 COVID-19 hospitalisations among the general population [17]. The present study found 3,622 total COVID hospitalisations in VIC, of which 210 were aged care resident hospitalisations. The Victorian study found high hospitalisation and case fatality rate among aged-care residents and suggested that 84% of hospitalised cases and 81% of deaths were aged-care residents [17]. Our study also found that 88.2% (210/238) of the total hospitalised cases are from VIC.

Contrary to the Victorian study, the present study found that only 10.5% (210/1998) residents and 17.8% (3,622/20,345) were hospitalised. Considering the reported COVID notifications in VIC, only 10.5% of the total infected residents and 17.8% of the total VIC population with COVID-19 were hospitalised. 45.8% (27/59) of the total infected residents were hospitalised in NSW. The case-by-case hospital transfer policy for COVID-infected residents could have led to low resident hospitalisation rates (3.14 per 100,000 population) in the VIC. We cannot conclude the same for NSW as all residents were hospitalised during the initial weeks of the outbreak, after which a case-by-case policy was adopted. NSW also allowed hospital-in-home programs in some residential facilities, which could have also affected the hospitalisation rates of infected residents [46].

Residential aged-care facilities are designed to be home-like spaces for the aged-care residents living in these facilities [20]. During the early wave of the COVID-19 pandemic, these facilities faced numerous challenges due to a lack of proper isolation spaces for the infected residents, communal living, PPE shortages and a lack of trained staff in infection control measures [54]. These facilities were not deemed suitable for treating viral respiratory infections like COVID-19; hence hospital-in-home programs could not meet the healthcare needs of the vulnerable aged-care residents or prevent spread in an outbreak situation [20]. Hospital-in-home failed to meet the challenges of the COVID-19 pandemic, and planning for other models of care in case of another pandemic is needed. Generally, hospitals are considered more suitable than RACFs due to high infection control protocol and better-trained staff. However, during the pandemic, hospitals struggled due to a sudden increase in COVID cases, other hospital admissions, lack of PPE supply and staff shortage [55]. The lessons learned during the pandemic were that RACFs and hospitals should improve their infrastructure, upskill their staff with infection control training, have a sufficient supply of PPE and have access to trained staff to reduce the risk of mortality and improve the survival rates of the aged-care residents.

The major limitation of this study was the use of open-source data, as the data generated from open sources cannot easily be verified. Open-sourced data obtained for this study may vary depending on each state’s data publishing policies. The official surveillance reports released by the Australian DoH did not include hospitalisation data; hence we cannot confirm if we have over or under-reported the resident hospitalisations owing to COVID infection. We cannot confirm if there were any duplications of cases as demographic details of the infected residents and staff were not mentioned in the official surveillance reports. However, our data was similar to later official reports. Another limitation of this paper is that there were no official publicly available data on aged-care resident hospitalisations for COVID-19 and other medical conditions, and the majority of the media reports/RACF blogs focussed on COVID hospitalisations of the aged-care residents during the pandemic; hence we could not determine the impact of hospital transfer policies for other medical conditions. This study was in the first year of the pandemic, and does not reflect the higher transmissibility of the Omicron sub-variants.

## Conclusion

We found high infection rates and increased risk of COVID-19 infection among aged-care residents and ACWs in the RACFs in Australia. High community transmission aggravated the COVID-19 situation in the facilities despite following IPC guidelines. We also found a low hospitalisation rate of aged-care residents across Australia which could have been due to the case-by-case policy for hospital transfer of residents; hence we recommend hospital transfer of the infected aged-care residents to reduce the risk of COVID-19 transmission to the remaining residents and for improving their survival rates. We suggest hospitals and RACFs consider improving their infrastructure, upskilling staff, increasing staff intake and ensuring an adequate supply of PPE and other facilities to prepare for the risk of a pandemic in future. We further recommend an epidemiological study that investigates the impact of Omicron variants on RACFs across Australia and impact of hospitalisation policies on this vulnerable population.

### Electronic supplementary material

Below is the link to the electronic supplementary material.

Supplementary Material 1

## Data Availability

The datasets used and analysed during the current study are available from the corresponding author on reasonable request.COVID-19 weekly reports are publicly available under COVID-19 outbreaks in Australian residential aged care facilities collection, https://www.health.gov.au/resources/collections/covid-19-outbreaks-in-australian-residential-aged-care-facilities. Aged-care resident population data and aged-care work force census datasets used for analyses are available from: https://www.gen-agedcaredata.gov.au/Resources/Access-data/2020/October/Aged-care-data-snapshot%E2%80%942020 and https://gen-agedcaredata.gov.au/Resources/Reports-and-publications/2021/October/2020-Aged-Care-Workforce-Census-Report. Australian population data is available at https://www.abs.gov.au/statistics/people/population/national-state-and-territory-population/jun-2020 and Australian death statistics is available at https://www.abs.gov.au/statistics/people/population/deaths-australia/2020.

## Abbreviations

ABS: Australian Bureau of Statistics
ACT: Australian Capital Territory
ACW: Aged-care worker
AIHW-GEN: Australian Institute of Health & Welfare
CDNA: Communicable Diseases Network Australia
CFR: Case fatality rate
DHAC: Department of Health and Aged-Care
DoH: Department of Health
HR: Hospitalisation rate
IR: Incidence rate
NSW: New South Wales
NT: Northern Territory
OR: Odds ratio
PCR: Polymerase Chain Reaction
PPE: Personal protective equipment
QLD: Queensland
RACF: Residential aged-care facility/facilities
RAT: Rapid Antigen Test
RR: Risk ratio
SA: South Australia
TAS: Tasmania
UK: United Kingdom
VIC: Victoria
WA: Western Australia
GP: General Practitioner

## References

[1] Dong E, Du H, Gardner L (2020). An interactive web-based dashboard to track COVID-19 in real time. Lancet Infect Dis.

[2] Onder G, Rezza G, Brusaferro S (2020). Case-fatality rate and characteristics of patients dying in relation to COVID-19 in Italy. JAMA.

[3] Yanez ND, Weiss NS, Romand JA, Treggiari MM (2020). COVID-19 mortality risk for older men and women. BMC Public Health.

[4] Burton JK, Bayne G, Evans C, Garbe F, Gorman D, Honhold N (2020). Evolution and effects of COVID-19 outbreaks in care homes: a population analysis in 189 care homes in one geographical region of the UK. Lancet Healthy Longev.

[5] Gilbert GL (2020). COVID-19 in a Sydney nursing home: a case study and lessons learnt. Med J Aust.

[6] Jordan RE, Adab P, Cheng KK (2020). Covid-19: risk factors for severe disease and death. BMJ.

[7] Viray P, Low Z, Sinnappu R, Harvey PA, Brown S (2021). Residential aged care facility COVID-19 outbreaks and magnitude of spread among residents: observations from a victorian residential in-reach service. Intern Med J.

[8] Dosa D, Jump RLP, LaPlante K, Gravenstein S (2020). Long-term care Facilities and the Coronavirus Epidemic: practical guidelines for a Population at Highest Risk. J Am Med Dir Assoc.

[9] Oran DP, Topol EJ (2020). Prevalence of asymptomatic SARS-CoV-2 infection: a narrative review. Ann Intern Med.

[10] Kimball A, Hatfield KM, Arons M, James A, Taylor J, Spicer K (2020). Asymptomatic and presymptomatic SARS-CoV-2 infections in residents of a long-term care skilled nursing facility - King County, Washington, March 2020. MMWR Morb Mortal Wkly Rep.

[11] Gan JM, Kho J, Akhunbay-Fudge M, Choo HM, Wright M, Batt F (2021). Atypical presentation of COVID-19 in hospitalised older adults. Ir J Med Sci.

[12] Lai E, Tan HY, Kunasekaran M, Chughtai AA, Trent M, Poulos C (2020). Influenza vaccine coverage and predictors of vaccination among aged care workers in Sydney Australia. Vaccine.

[13] Quigley A, Stone H, Nguyen PY, Chughtai AA, MacIntyre CR (2022). COVID-19 outbreaks in aged-care facilities in Australia. Influenza Other Respir Viruses.

[14] Royal. Commission into Aged Care Quality and Safety. Interim report: neglect. 2019.

[15] Australian Government Department of Health. CDNA national guidelines for the prevention, control and public health management of COVID-19 outbreaks in residential care facilities in Australia. Canberra: Australian Government Department of Health. ; 2020. Available from: https://www.health.gov.au/sites/default/files/documents/2022/02/cdna-national-guidelines-for-the-prevention-control-and-public-health-management-of-covid-19-outbreaks-in-residential-care-facilities-in-australia.pdf.

[16] Aitken GE, Holmes AL, Ibrahim JE (2021). COVID-19 and residential aged care: priorities for optimising preparation and management of outbreaks. Med J Aust.

[17] Victorian Department of Health COVID-19 writing group (2021). Population-based analysis of the epidemiological features of COVID-19 epidemics in Victoria, Australia, January 2020 - March 2021, and their suppression through comprehensive control strategies. Lancet Reg Health West Pac.

[18] Ayouni I, Maatoug J, Dhouib W, Zammit N, Fredj SB, Ghammam R (2021). Effective public health measures to mitigate the spread of COVID-19: a systematic review. BMC Public Health.

[19] Department of Health & Social Care, Public Health England, Care Quality Commission and, England NHS. Admission and care of residents in a care home during COVID-19 2020 Available from: https://www.gov.uk/government/publications/coronavirus-covid-19-admission-and-care-of-people-in-care-homes/coronavirus-covid-19-admission-and-care-of-people-in-care-homes.

[20] Royal Commission into Aged Care Quality and Safety. Aged care and COVID-19: a special report. 2020 Available from: https://agedcare.royalcommission.gov.au/sites/default/files/2020-10/aged-care-and-covid-19-a-special-report.pdf.

[21] Australian Government Department of Health. COVID-19 Outbreaks in Residential Care Facilities - National Guidelines for the Prevention, Control and Public Health Management of COVID-19 Outbreaks in Residential Care Facilities 2022 [Available from: https://www.health.gov.au/resources/publications/cdna-national-guidelines-for-the-prevention-control-and-public-health-management-of-covid-19-outbreaks-in-residential-care-facilities-in-australia.

[22] Australian Institute of Health and Welfare. Australia’s hospitals at a glance Canberra: Australian Institute of Health and Welfare; 2022 [cited 2023 May 22]. Available from: https://www.aihw.gov.au/reports/hospitals/australias-hospitals-at-a-glance.

[23] Australia Government. Department of Health and Aged Care. COVID-19 outbreaks in Australian residential aged care facilities Canberra: Department of Health and Aged Care; 2020 Available from: https://www.health.gov.au/resources/collections/covid-19-outbreaks-in-australian-residential-aged-care-facilities.

[24] Tasmanian Government. Department of Health. TAS News Releases Tasmania: Department of Health (Tasmania). ; 2020 Available from: https://www.health.tas.gov.au/news/media-releases.

[25] NSW Government. NSW Health. Media Releases Sydney: NSW Health. ; 2020 Available from: https://www.health.nsw.gov.au/news/Pages/2023-nsw-health.aspx.

[26] Victoria State Government. Department of Health. Media releases Melbourne: Victoria State Government, Department of Health;2020. Available from: https://www.health.vic.gov.au/media-centre/media-releases.

[27] Government ACT. COVID-19: News updates Canberra: ACT Government; 2020 Available from: https://www.covid19.act.gov.au/updates/news-updates.

[28] Government of Western Australia. Department of Health. Latest News: Media Releases Perth: WA Health. ; 2020 Available from: https://www.health.wa.gov.au/News/Media-releases-listing-page.

[29] Northern Territory Government. NT Health. Coronavirus (COVID-19) Northern Territory. Darwin: NTG. ; 2020 [Available from: https://health.nt.gov.au/covid-19].

[30] Australia Government. Department of Health. Managing a COVID-19 outbreak in residential aged care Canberra: Australia Government Department of Health; 2021. Available from: https://www.health.gov.au/topics/aged-care/advice-on-aged-care-during-covid-19/managing-a-covid-19-outbreak-in-residential-aged-care.

[31] St. Louis Aged Care. COVID 19 Statement Adelaide: St. Louis Aged Care; 2020 Available from: https://www.stlouisagedcare.com.au/blog/covid-19-statement/].

[32] Elizabeth Daoud. Coronavirus in Australia: Death toll rises as aged care resident from Dorothy Henderson Lodge dies from COVID-19. 2020. Sydney: 7news; 2020 Available from: https://7news.com.au/lifestyle/health-wellbeing/coronavirus-in-sydney-another-aged-care-resident-from-the-dorothy-henderson-lodge-dies-from-covid-19-c-959773].

[33] Australian Government. Department of Health. Coronavirus (COVID-19) case numbers and statistics Canberra: Australian Government. Department of Health 2021 Available from: https://www.health.gov.au/health-alerts/covid-19/case-numbers-and-statistics].

[34] Australian Government. Department of Health and Aged Care. Aged-care data snapshot – 2020 Canberra: AIHW. ; 2020. Available from: https://www.gen-agedcaredata.gov.au/Resources/Access-data/2020/October/Aged-care-data-snapshot-2020.

[35] Australia Government. Department of Health. 2020 Aged Care Workforce Census Canberra: Australian Government. Department of Health; 2021 Available from: https://www.health.gov.au/resources/publications/2020-aged-care-workforce-census.

[36] Australian Bureau of Statistics. National, state and territory population Canberra: ABS. ; 2020 Available from: https://www.abs.gov.au/statistics/people/population/national-state-and-territory-population/jun-2020.

[37] Australian Bureau of Statistics, Deaths A, Canberra ABS. ; 2020 Available from: https://www.abs.gov.au/statistics/people/population/deaths-australia/2020].

[38] Rebecca, Le May. Infected worker sparks aged care lockdown; 2020. Available from: https://www.canberratimes.com.au/story/6683365/infected-worker-sparks-aged-care-lockdown.

[39] von Elm E, Altman DG, Egger M, Pocock SJ, Gøtzsche PC, Vandenbroucke JP (2008). The strengthening the reporting of Observational Studies in Epidemiology (STROBE) statement: guidelines for reporting observational studies. J Clin Epidemiol.

[40] StataCorp. Stata Statistical Software: Release 16. TX: StataCorp LLC ; 2019.

[41] Ministry of Health. Guiding principles for safe and efficient admissions into Residential Aged Care Facilities and transfers to hospital during the COVID-19 pandemic 2020 Available from: https://agedcare.royalcommission.gov.au/system/files/2020-08/RCD.9999.0381.0086.pdf.

[42] Victoria State Government. Department of Health and Human Services. Admission and transfer to and from Residential Aged Care Facilities and hospitals. Coronavirus (COVID-19) advice. Melbourne: DHHS. ; 2020 Available from: https://www.dhhs.vic.gov.au/sites/default/files/documents/202011/Admission and transfer to and from Residential Aged Care Facilities and Hospitals.

[43] Queensland Government. COVID-19 Outbreak Management - Preparing and responding -Guidance for Residential Aged Care Facilities in Queensland Brisbane: Queensland Health. ; 2020. Available from: https://www.health.qld.gov.au/__data/assets/pdf_file/0025/1004677/racf-covid19-outbreak-management-guidelines.pdf.

[44] Government of Western Australia. Department of, Health. COVID-19 information for aged care and community care providers Perth: WA Health; 2020 [Available from: https://www.health.wa.gov.au/Articles/A_E/Coronavirus/COVID19-information-for-Aged-Care-and-Community-Care-Providers.

[45] Office of Ageing Well. South Australian strategy for responding to COVID-19 residential aged care facilities Adelaide: SA Health. ; 2020. Available from: https://www.lasa.asn.au/wp-content/uploads/2020/10/SA-COVID-19-Strategy-for-RACF_V2.pdf.

[46] Tasmania Government. Department of Health. COVID-19, Influenza, Respiratory Syncytial Virus, and Other Acute Respiratory Infection Outbreaks in Residential Aged Care Facilities Tasmania: TAS Health; 2020. Available from: https://www.health.tas.gov.au/sites/default/files/2022-12/covid-19_outbreaks_in_residential_aged_care_facilities_toolkit_to_support_planning_preparedness_and_response.pdf.

[47] Hashan MR, Smoll N, King C, Ockenden-Muldoon H, Walker J, Wattiaux A (2021). Epidemiology and clinical features of COVID-19 outbreaks in aged care facilities: a systematic review and meta-analysis. EClinicalMedicine.

[48] Australia Government. Department of Health. Prevent and prepare for COVID-19 in residential aged care Canberra: Australian Government. Department of Health.; 2020 [Available from: https://www.health.gov.au/topics/aged-care/advice-on-aged-care-during-covid-19/prevent-and-prepare-for-covid-19-in-residential-aged-care.

[49] Australian Government. Department of Health. Information for aged care providers, workers and residents about COVID-19 vaccines Canberra: Australian Government. Department of Health ; 2020 Available from: https://www.health.gov.au/our-work/covid-19-vaccines/information-for-aged-care-providers-workers-and-residents-about-covid-19-vaccines.

[50] Price DJ, Shearer FM, Meehan MT, McBryde E, Moss R, Golding N et al. Early analysis of the australian COVID-19 epidemic. Elife. 2020; 9. 10.7554/eLife.58785 PMC7449695 32788039

[51] Hutchinson D, Williams H, Stone H. COVID-19 variants of concern in Australia, September 2020-April 2021. Global Biosecur. 2021; 3(1).

[52] Zhou B, Thao TTN, Hoffmann D, Taddeo A, Ebert N, Labroussaa F (2021). SARS-CoV-2 spike D614G change enhances replication and transmission. Nature.

[53] Royal Commission into Aged Care Quality and Safety. Hospitalisations in Australian Aged Care: 2014/15–2018/19 Canberra: Commonwealth of Australia. ; 2021. Available from: https://agedcare.royalcommission.gov.au/sites/default/files/2021-02/research-paper-18-hospitalisations-australian-aged-care.pdf.

[54] Gilbert L, Lilly A. Independent review: COVID-19 outbreaks in Australian residential aged care facilities. 2021.

[55] Ayton D, Soh SE, Berkovic D, Parker C, Yu K, Honeyman D (2022). Experiences of personal protective equipment by australian healthcare workers during the COVID-19 pandemic, 2020: a cross-sectional study. PLoS ONE.

